# Endothelial cell ferroptosis influences IDH wild-type glioblastoma growth in recurrent glioblastoma multiforme patients

**DOI:** 10.1590/1414-431X2024e13961

**Published:** 2024-07-08

**Authors:** Bo Liang, Xinghuan Ding, Siyuan Yang, Enshan Feng

**Affiliations:** 1 Department of Neurosurgery, Beijing Ditan Hospital, Capital Medical University, Beijing, China; 2 Laboratory of Infectious Diseases Center, Beijing Ditan Hospital, Capital Medical University, Beijing, China

**Keywords:** Glioblastomas, Single-cell sequencing, NOX4, Ferroptosis, Endothelial cells

## Abstract

Glioblastomas are known for their poor clinical prognosis, with recurrent tumors often exhibiting greater invasiveness and faster growth rates compared to primary tumors. To understand the intratumoral changes driving this phenomenon, we employed single-cell sequencing to analyze the differences between two pairs of primary and recurrent glioblastomas. Our findings revealed an upregulation of ferroptosis in endothelial cells within recurrent tumors, identified by the significant overexpression of the *NOX4* gene. Further analysis indicated that knocking down NOX4 in endothelial cells reduced the activity of the ferroptosis pathway. Utilizing conditioned media from endothelial cells with lower ferroptosis activity, we observed a decrease in the growth rate of glioblastoma cells. These results highlighted the complex role of ferroptosis within tumors and suggested that targeting ferroptosis in the treatment of glioblastomas requires careful consideration of its effects on endothelial cells, as it may otherwise produce counterproductive outcomes.

## Introduction

Glioblastoma multiforme (GBM), the most prevalent form of primary malignant central nervous system tumors, poses a significant threat to patient survival and quality of life. Constituting approximately 80% of all tumors of the central nervous system, its complexity and high recurrence rate contribute to poor therapeutic outcomes, resulting in dismal prognoses for affected individuals (1). Notably, the three-year survival rate for patients with gliomas is below 20%, and even with comprehensive treatment regimens, the average survival duration post-recurrence is merely 12 to 15 months (2).

Most brain gliomas harbor mutations in isocitrate dehydrogenase (IDH) 1 and 2, a characteristic that has significant implications for the disease's pathogenesis and patient prognosis (3,4). However, a subset of gliomas exhibits wild-type IDH, which is often associated with a worse prognosis (5). The recurrent nature of gliomas is a major challenge; recurrent tumors tend to be more aggressive than the primary tumor, highlighting the need for innovative treatment strategies.

The tumor microenvironment (TME) of GBM has been identified as a key factor influencing treatment efficacy (6,7). The TME, comprising various cell types such as microglia, macrophages, astrocytes, oligodendrocytes, neurons, neural progenitor cells, extracellular matrix components, pericytes, and endothelial cells, forms a complex network (8). This network facilitates interactions between tumor and non-tumor cells, creating a local environment that supports tumor cell growth, invasiveness, cell death resistance, and/or therapeutic resistance, as well as immune evasion (8,9).

Therefore, alterations in the tumor microenvironment and changes in cellular interactions are believed to contribute to the enhanced drug resistance and invasive capabilities observed in gliomas post-recurrence (10). This underscores the urgent need for a deeper understanding of the TME's role in glioma progression and recurrence, which could unlock new pathways for targeted therapies and improve patient outcome. However, the identification of specific cellular components responsible for these changes, as well as the determination of altered intercellular interactions remain challenging through bulk omics approaches.

Single-cell sequencing, on the other hand, offers a precise means to distinguish changes in different cell populations within the tumor and the gene expression variations across cell types (11-14). In this study, we selected primary and recurrent tumor tissues from two patients with IDH wild-type gliomas. By conducting single-cell sequencing analysis on these tumor tissues, we aimed to uncover the changes occurring within the recurrent tumors of the same patient under the same treatment regimen.

Our study particularly focuses on the signaling pathways related to ferroptosis between primary and recurrent tumors. Ferroptosis is the increased susceptibility of recurrent glioblastoma to an iron-dependent type of cell death (15). Unlike apoptosis, autophagy, necrosis, or pyroptosis, ferroptosis is characterized by an increase in lipid peroxidation, leading to mitochondrial atrophy and an increase in mitochondrial membrane density, thereby causing an accumulation of reactive oxygen species (ROS) (2,16-18). The increase of ferroptosis in gliomas is associated with tumor growth slowdown, potentially offering favorable therapeutic outcomes (19). Furthermore, genetic features associated with ferroptosis can aid in predicting the risk of GBM and evaluating its prognosis (20).

Within the ferroptosis signaling pathway, the *NOX4* gene, belonging to the NADPH oxidase family, plays a crucial role in converting superoxide into H_2_O_2_. This gene is overexpressed in gliomas (21,22). Our investigation into these pathways aimed to provide a deeper understanding of the mechanisms behind glioma recurrence and ferroptosis, offering new insights into potential therapeutic targets and prognostic markers for GBM.

In our research, we validated the upregulation of *NOX4* expression on endothelial cells in the recurrent samples through single-cell sequencing. Furthermore, by knocking down *NOX4* on the endothelial cells, we found lower proliferation rates of glioma cells in the co-culture system, indicated the complex effect of ferroptosis in GBM tumor.

## Material and Methods

### Dataset

**Figure 1:**
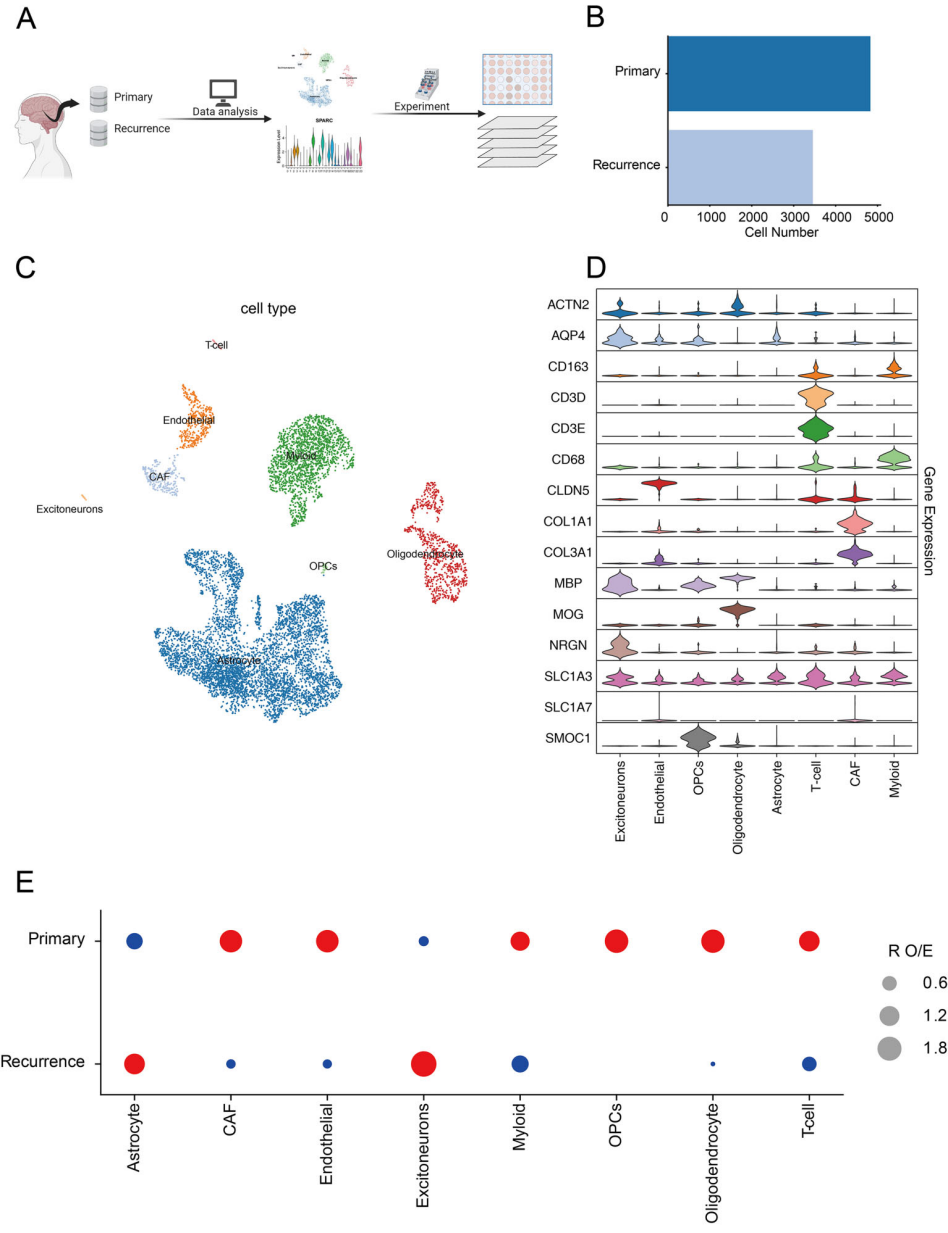
**A**, Data analysis process. **B**, Cell number for single-cell sequence in primary and recurrent glioblastoma multiforme patient samples. **C**, Uniform manifold approximation and projection of different cell types. **D**, Violin plots of biomarker expression across different cell types. **E**, Relative order of events (ROE) analysis of different cell types in primary and recurrent tumor samples. CAF: cancer-associated fibroblast; OPCs: oligodendrocyte precursor cells.

In this study, we utilized a publicly available single-cell RNA sequencing dataset (scRNA: GEO, GSE131907). Four samples (JK136, JK142, JK196, JK202) were used, including 2 primary and 2 recurrent samples from 2 patients (Figure 1A and B).

### Data preprocessing and quality control

#### Data quality control

We conducted data quality control using the Scanpy (V1.9) toolkit (https://scanpy.readthedocs.io/en/1.9.x/), filtering out cells with gene counts less than 800 and more than 5000. The minimum read counts were set to less than 1000 and the maximum to more than 20000, with a mitochondrial gene cutoff value of 20%. This resulted in 8285 cells.

#### Data processing

Normalization was performed using the scanpy function sc.pp.normalize_per_cell. We identified the top 2000 highly variable genes (sc.pp.highly_variable_genes) for PCA analysis (sc.tl.pca). Data integration and batch correction were carried out using harmony (sc.external.pp.harmony_integrate). Subsequently, uniform manifold approximation and projection (UMAP) dimensionality reduction and Louvain clustering analysis were performed.

### Cell lines and cell culture

Human umbilical vein endothelial cells (HUVECs) and U251 cells were purchased from the Cell Resource Center, Peking Union Medical College (PCRC). U251 cells were cultured in complete medium consisting of DMEM supplemented with 10% FBS and 1% penicillin, and HUVECs were cultured using complete human umbilical vein endothelial cell medium (Starfish Bio, PAHX-G131, China). U251 and HUVEC were cultured overnight in a 37°C incubator with 5% CO_2_.

#### Cell transfection

HUVEC cells were digested with 0.25% trypsin-EDTA to form a single-cell suspension, washed, resuspended in medium, and counted. *NOX4* gene siRNA and control siRNA (Table 1) at concentrations of 50 nM were added, and transfection was carried out using RNAimax (13778030, Thermo Scientific, USA). Cells were then returned to the incubator for downstream experiments. The successfully transfected HUVECs were renamed as HUVEC-*NOX4*-Si-1 and HUVEC-siCtrl.

**Table 1:** siRNA design used in the study.

Gene ID	Sequence	
	sense (5'-3')	antisense (5'-3')
*NOX4* si-1	GGAUAAAAGCAGAACAUUCdCdA	GAAUGUUCUGCUUUUAUCCdAdA
siCtrl	UUCUCCGAACGUGUCACGUdTdT	ACGUGACACGUUCGGAGAAdTdT

### RNA extraction, reverse transcription, and Q-PCR

#### RNA isolation from transfected HUVECs

Forty-eight hours post-transfection, HUVECs were harvested and digested with trypsin. Cells were then centrifuged at 2,000 *g* for 5 min at 4°C, and the cell pellet was resuspended with Trizol. Chloroform (200 μL) was added and cells were left at room temperature for 10 min, then centrifuged at 4°C and 12,000 *g* for 15 min. The upper aqueous phase was carefully transferred to a new 1.5-mL centrifuge tube. An equal volume of pre-cooled isopropanol was added to the aqueous phase, and then incubated at 4°C for 10 min. The samples were centrifuged again at 4°C and 12,000 *g* for 12 min, and the RNA pellet was collected, washed with 75% ethanol, and dissolved with RNase-free water.

Reverse transcription was applied following the user guide of the ReverTra Ace qPCR RT kit (FSQ-201, TOYOBO, Japan), and qPCR was applied using Takara SYBR Master Mixture (RR420, Takara, Japan) for the quality analysis of ferroptosis-related genes *NOX4*, *GPX4*, *SLC7A11* and using *β-actin* as internal control. The primer set of related genes is shown in Table 2.

**Table 2:** qPCR primers used in the study.

Gene	Sequence
*NOX4*	
Forward	CAGTCTTTGACCCTCGGTCC
Reverse	GAGCCAGATGAACAGGCAGA
*GPX4*	
Forward	GAGGCAAGACCGAAGTAAACTAC
Reverse	CCGAACTGGTTACACGGGAA
*SLC7A11*	
Forward	TGTGTGGGGTCCTGTCACTA
Reverse	CAGTAGCTGCAGGGCGTATT
*β-actin*	
Forward	CATGTACGTTGCTATCCAGGC
Reverse	CTCCTTAATGTCACGCACGAT

### Cell proliferation assay

The viability of glioma cells was assessed using the CCK8 assay (ab228554, Abcam, USA). U251 cells were co-cultured for 24 h with lysates from HUVEC-siCtrl and HUVEC-*NOX4*si-1 cells, and the changes in the viability of U251 cells were then measured using the CCK8 method.

### Statistical analysis

Normally distributed data were compared by unpaired Student’s *t*-test for two groups comparisons, abnormally distributed data were compared by Mann-Whitney test. P-values <0.05 were considered significantly different. GraphPad Prism (version 5.0, USA) and SPSS software (version 23.0, IBM, USA) were used for statistical analyses.

## Results

In clinical treatment, glioblastomas often exhibit increased aggressiveness and faster growth rates upon recurrence. To address the difference between primary and recurrent tumor, we selected two pairs of primary and recurrent IDH wild-type glioblastoma samples from a single-cell sequencing database (GSE131907) of glioblastoma patients. In each pair, the primary and recurrent tumors originated from the same patient to minimize the interference of other factors.

Initially, we employed the UMAP (uniform manifold approximation and projection) method to distinguish various cell types within glioblastoma, including excitatory neurons, endothelial cells, oligodendrocyte precursor cells (OPCs), oligopoly cells, astrocytes, T cells, cancer-associated fibroblasts (CAFs), and myeloid cells (Figure 1C). The biomarkers used for these cellular classifications and the percentage of those cells in different groups are presented in Table 3.

**Table 3:** Cellular classification in primary and recurrent glioblastoma multiforme samples.

Cells	Biomarker	Percentage in primary tumor	Percentage in recurrent tumor
Excitatory neurons	NRGN, SLC1A7	0.08%	0.66%
Endothelial cells	DCN, ACTN2	7.18%	0.92%
OPCs	SMOC1	0.65%	0
Oligopoly cells	MOG, BMP	15.36%	0.28%
Astrocytes	AQP4, SCL1A3	48.37%	79.59%
T-cells	CD3D, CD3E	0.44%	0.20%
CAFs	COL1A1, COL3A1	4.87%	0.66%
Myeloid cells	CD68, CD163	23.04%	17.65%

OPCs: oligodendrocyte precursor cells; CAFs: cancer-associated fibroblasts.

Analysis of the relative order of events (ROE) in cell proportions revealed an increased expression of astrocytes in the recurrent samples, whereas the levels of fibroblasts, endothelial cells, and other types were relatively decreased (Figure 1D and E).

**Figure 2:**
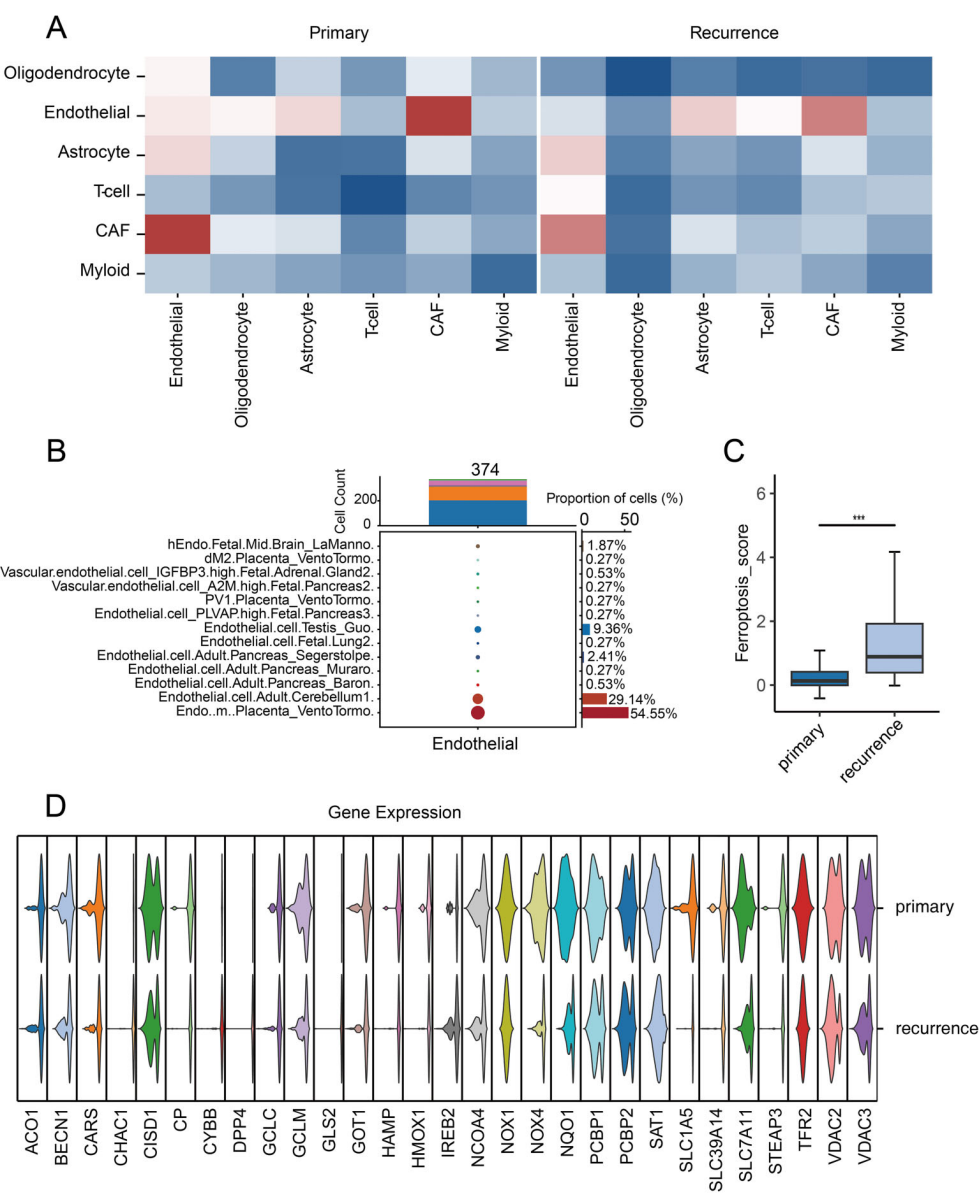
**A**, Cell communication analysis showed that endothelial cells had relatively strong communication with other cells in the immune microenvironment. **B**, Cell origin analysis of endothelial cells in the samples. **C**, Ferroptosis pathway score in primary and recurrent tumors. ***P<0.001, Student’s *t*-test. **D**, Violin plots of ferroptosis pathway related genes. CAF: cancer-associated fibroblast.

We then analyzed the cell communication among different cell types in primary and recurrent samples. We found that endothelial cells had strong communication with other tumor, stromal, and immune cells in both primary and recurrent tumors (Figure 2A). This finding suggested that endothelial cells may play an important role in the growth regulation of primary and recurrent GBMs. We further analyzed the origin of endothelial cells in the samples, and found that 54.55% of them originated from placental endothelial cells, while 29.14% came from cerebellar endothelial cells (Figure 2B).

To focus on endothelial cells, we used the ferroptosis score system to evaluate the activation of the ferroptosis pathway. The analysis showed a significant increase in the activation of ferroptosis in the endothelial cells of the recurrent tumor (Figure 2C). Through gene set heterogeneity analysis, we confirmed that the ferroptosis pathway in endothelial-like cells was highly expressed in recurrent tumors. The expression levels of genes involved in oxidation-reduction processes, such as *CISD1*, *NOX4*, *HMOX1*, *NQUO1*, *CYBB*, DPP4, and NOX1, were higher in recurrent tumors compared to primary tumors (Figure 2D). *NOX4* showed the most significant difference.

**Figure 3:**
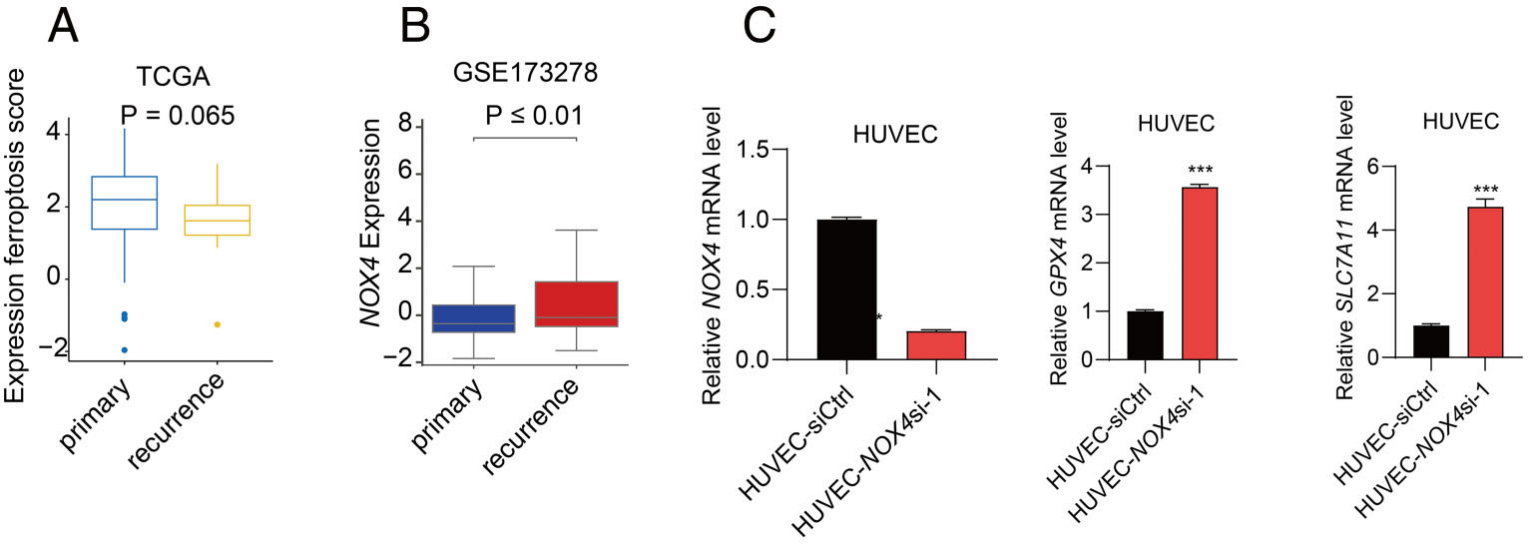
**A**, Ferroptosis pathway expression analysis based on TCGA RNAseq dataset. **B**, *NOX4* expression in endothelial cells in primary and recurrence glioblastoma multiforme (GBM) in the GSE173278 dataset. **C**, *NOX4* expression in human umbilical vein endothelial cells (HUVEC) post-RNAi transfection. GPX4 gene expression in control HUVEC-siCtrl and *NOX4* knockdown cells (HUVEC-*NOX4*si-1). SLC7A11 gene expression in control HUVEC-siCtrl and HUVEC-*NOX4*si-1 cells. Data are reported as median and interquartile range (**A** and **B**) and mean and SD (**C**). ***P<0.001, Mann-Whitney test and Student’s *t*-test.

However, when comparing the expression of ferroptosis-related pathway genes in primary and recurrent gliomas using the TCGA brain glioma RNAseq database (https://portal.gdc.cancer.gov/projects/TCGA-GBM), no significant difference was observed (Figure 3A, Wilcoxon analysis, P=0.065). This suggested that in TCGA samples predominantly composed of epithelial cells, changes in endothelial cells are averaged out and undetectable. This further highlighted the necessity of using single-cell omics methods in tumor research for precise analysis of different cell types. In order to overcome the limited samples, we added another 12 GBM single-cell sequencing samples from another dataset (GSE173278) to validate our findings. *NOX4* was also significantly up-regulated in recurrence endothelial cells compared with the primary endothelial cells (Figure 3B, P-value <0.01). This result strengthened our finding across a broader range of GBM.

To further validate whether the upregulation of the ferroptosis pathway in endothelial cells is responsible for the distinct growth characteristics, increased invasiveness, and accelerated growth rate observed in recurrent tumors compared to primary tumors, we utilized human umbilical vein endothelial cells (HUVECs) to simulate endothelial cells in GBM. We knocked down the expression of the *NOX4* gene in these cells using RNA interference. The resulting *NOX4* knockdown HUVEC cells (HUVEC-*NOX4*si-1) showed a significant reduction in *NOX4* gene expression compared to the Control cells (HUVEC-siCtrl) (Figure 3C). To further investigate the impact of *NOX4* knockdown on the ferroptosis pathway, we examined the expression of ferroptosis-related genes *GPX4* and *SLC7A11*.

*GPX4* primarily functions to reduce lipid peroxides, thereby protecting cells from oxidative stress damage. It uses glutathione (GSH) as a cofactor to directly reduce lipid peroxides, preventing the chain reaction of lipid peroxidation. The upregulation of GPX4 enhances the cell's ability to counteract lipid peroxidation, and is usually associated with the inhibition of ferroptosis (17).

The *SLC7A11* gene encodes a subunit of the system Xĉ- transporter and plays a significant role in ferroptosis. When the expression or activity of *SLC7A11* is inhibited, it leads to a decrease in GSH levels, thereby weakening GPX4 function and making cells more susceptible to ferroptosis. Therefore, the activity of SLC7A11 is crucial for maintaining the cell's anti-ferroptotic state (23,24).

We found that in HUVEC-*NOX4*si-1 cells, where *NOX4* gene was knocked down, there was a significant increase in the expression of *GPX4* and *SLC7A11* genes compared to the control HUVEC-siCtrl cells (Figure 3C). This indicated that *NOX4* knockdown significantly inhibited the ferroptosis pathway in HUVEC cells.

**Figure 4:**
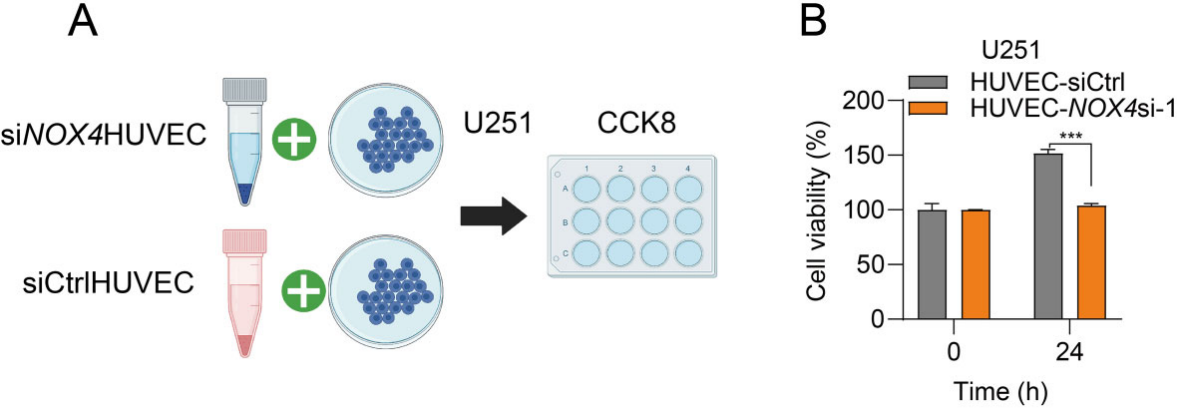
**A**, Experimental flowchart illustrating the co-culture of conditioned media from human umbilical vein endothelial cells (HUVEC)-*NOX4*si-1 (*NOX4* knockdown) cells and HUVEC-siCtrl cells (control) with U251 cells, followed by the assessment of U251 cell proliferation changes using the CCK8 assay. **B**, U251 cell proliferation significantly decreased when co-cultured with HUVEC-*NOX4*-si-1 cell conditioned media. Data are reported as mean and SD; ***P<0.001, Student’s *t*-test.

Furthermore, we co-cultured the conditioned media from HUVEC-siCtrl and HUVEC-*NOX4*si-1 cells with glioma U251 cells. The results showed that the conditioned media from HUVEC-*NOX4*si-1 cells significantly inhibited the proliferation of U251 cells (Figure 4A and B). This finding links the reduction of ferroptosis in endothelial cells to the proliferation of glioma cells.

These results showed that ferroptosis of endothelial cells can influence tumor cell growth and may play a key role in the increase of the tumor growth rate in recurrent GBM tumors, which indicated that using single-cell sequence may have clinical value.

## Discussion

In this research, we employed single-cell sequencing to delineate the differences between primary and recurrent IDH-negative glioblastomas. A notable finding was the enhanced communication between endothelial cells and other cell types in both primary and recurrent tumors. Particularly important was the significant activation of the ferroptosis signaling pathway in endothelial cells of recurrent tumors compared to primary ones. This activation was marked by the upregulation of redox-related genes, notably *CISD1*, *NOX4*, *HMOX1*, *NQO1*, *CYBB*, *DPP4*, and *NOX1*, with *NOX4* showing the most significant increase. This phenomenon was not observed in the TCGA database, likely due to the limitations of RNAseq data, which averages the expression of various cell types within the tumor, obscuring the distinct gene expression profiles of different cell types. Single-cell sequencing, by providing detailed genomic characteristics of different cell subtypes, proves more valuable in such research.

Further investigation into the role of *NOX4* overexpression in endothelial cells revealed that knocking down *NOX4* in HUVEC endothelial cells led to a decrease of ferroptosis activity. Co-culturing these *NOX4*-knockdown HUVECs with U251 glioblastoma cells resulted in decreased proliferation of cancer cells. suggesting a complex interplay between endothelial cells and tumor growth. Enhanced endothelial cell ferroptosis can promote the proliferation of glioblastoma tumor cells, which is consistent with our findings of higher ferroptosis pathway expression in endothelial cells from clinical recurrent samples.

All these findings were observed in *in vitro* cell lines, and the reliance on cell culture models, such as HUVEC and U251 cells, may not adequately mimic the *in vivo* tumor microenvironment, potentially limiting the clinical applicability of the findings. In order to overcome the limited samples and the difference between tumor microenvironment and cell line, we expanded to another 12 GBM single-cell sequencing samples from another dataset (GSE173278) to validate our findings. *NOX4* was also significantly up-regulated in recurrence endothelial cells compared with primary endothelial cells (Figure 3B, P-value <0.01). This result strengthened our finding across a broader range of GBM.

Ferroptosis has been identified as a potential therapeutic target for GBM (25,26). However, our findings suggested a paradoxical mechanism in recurrent IDH-negative glioblastomas, where upregulation of the ferroptosis pathway in endothelial cells may enhance tumor cell proliferation and invasiveness. This underscored the importance of considering the impact of ferroptosis-based therapies on the tumor microenvironment, as they may have opposing effects on different cell types within GBM tumors. However, conventional pharmacological experiments often use CDX and PDX models, which have a lower stromal cell component (10-20%) compared to clinical tumors, limiting their ability to reflect the interactions with stromal cells (27,28). These models primarily validate the effects of drugs on tumor cells, potentially overlooking mechanisms involving stromal cells. The opposing effects of stromal cells in tumors may be missed during the pre-clinical development of a ferroptosis-related treatment, but will appear in clinical research, causing the failure of drug development.

In tumors like GBM, characterized by extensive communication among various cell types, changes in stromal cells can significantly alter tumor growth characteristics. Therefore, developing tumor transplantation models with a complete stromal cell population is crucial for the advancement of cancer therapies.

In conclusion, our study highlights the complexity of tumor-stroma interactions in GBM, particularly the role of endothelial cells and ferroptosis pathways. The findings call for a more nuanced approach in developing GBM therapies, considering the diverse cellular landscape and the intricate interplay between different cell types within the TME. Based on our findings of the role of ferroptosis in tumor progression, the importance of considering how ferroptosis-based therapies could impact the tumor microenvironment is underscored. Developing models that include a complete stromal cell population is crucial, as changes in these cells can significantly alter tumor growth characteristics and therapeutic responses in the future.
